# Artificial Intelligence in Eye Disease: A Systematic Review of Screening, Diagnosis, Prognosis, Treatment-Response Prediction, and Clinical Decision Support

**DOI:** 10.3390/jcm15207957

**Published:** 2026-10-14

**Authors:** Manal M. Alharbi, Emtenan M. Alharbi, Alanoud Suliman Alsalamah, Hanan Asiri, Najla Beati, Renad AlAtawi, Amr Arafat

**Affiliations:** 1 Department of Optometry, College of Applied Medical Sciences, King Saud University, Riyadh 11433, Saudi Arabia; 2 Department of Clinical Pharmacy, College of Pharmacy, King Saud University, Riyadh 11451, Saudi Arabia; 3 Research Department, Prince Sultan Cardiac Center, Ministry of Defense Health Services, Riyadh 12426, Saudi Arabia; 4 Research and Innovation Institute, Ministry of Defense Health Services, Riyadh 12426, Saudi Arabia; 5 College of Medicine, King Saud University, Riyadh 11472, Saudi Arabia

**Keywords:** artificial intelligence, ophthalmology, clinical management, treatment-response prediction, clinical decision support, fundus photography, optical coherence tomography, explainable artificial intelligence, systematic review

## Abstract

**Background/Objectives**: Artificial intelligence (AI) is expanding eye care beyond image-based detection toward prognosis, treatment-response prediction, monitoring, triage, and clinician-facing decision support. We evaluated AI across eye-disease screening, diagnosis, prognosis, treatment response, and decision support, emphasizing validation, explainability, and clinical readiness. **Methods**: This PROSPERO-registered review followed the Preferred Reporting Items for Systematic Reviews and Meta-Analyses (PRISMA) 2020 statement. Six databases plus supplementary routes identified English-language literature published from 1 January 2015 through 10 August 2026. Included studies underwent design-mapped Joanna Briggs Institute (JBI)/adapted-JBI item-level appraisal; heterogeneity precluded meta-analysis. **Results**: The evidence base comprised 270 studies. Diabetic retinopathy/diabetic macular oedema was the largest clinical area (82), followed by retinal/multi-disease AI (52), glaucoma (46), and age-related macular degeneration (AMD)/macular disease (29); convolutional neural network (CNN)/convolutional deep learning was the largest primary method group (136). External validation was confirmed in 130 studies, including 95 with a clinical/geographic/prospective/real-world external-validation component (86 clinical-only and nine mixed clinical + technical); 49 had a strict direct clinical comparator and 90 used explainability. Pediatric/pediatric-relevant evidence accounted for 30 studies, while explicit optometry/optometrist involvement appeared in only three. **Conclusions**: Ophthalmic AI spans diagnosis through clinical management, but translation remains limited by heterogeneous outcomes, incomplete independent validation, few direct comparators, and sparse evaluation of safety, workflow effects, and patient outcomes.

## 1. Introduction

Ophthalmic diseases contribute substantially to preventable visual impairment and health-system burden. Early detection, accurate classification, reliable prognostic assessment, and timely treatment decisions are essential for conditions such as diabetic retinopathy, glaucoma, age-related macular degeneration, pediatric refractive disorders, retinopathy of prematurity, and other retinal or ocular diseases [1,2].

Artificial intelligence (AI) is particularly relevant to eye care because ophthalmology is supported by structured, imaging-rich clinical data. Fundus photography, optical coherence tomography (OCT), optical coherence tomography angiography (OCTA), ultra-widefield imaging, visual-field testing, clinical records, and multimodal datasets can support lesion detection, disease grading and classification, screening triage, prognosis, and clinician-facing decision support [1,2,3].

The clinical landscape is moving beyond isolated single-task classifiers toward multimodal and clinically integrated systems. AI is increasingly evaluated not only for diagnostic accuracy but also for prognosis, treatment-response prediction, monitoring, triage, workflow efficiency, usability, and safety. Diagnostic performance alone does not guarantee clinical utility, equitable implementation, or safe integration into real-world care pathways [4]. Recent ophthalmic-AI literature therefore increasingly emphasizes external validation, implementation, regulatory readiness, and care-pathway integration [5,6,7,8,9].

Existing ophthalmic-AI reviews provide important but different perspectives. Srivastava et al. summarized artificial-intelligence and machine-learning applications across ophthalmology [10]; Costin et al. reviewed the advantages and limitations of AI in ophthalmology [11]; and Savastano et al. synthesized progress, challenges, and ethical implications [12]. More recent reviews have addressed narrower disease-, device-, or implementation-specific questions [5,6,7,8,9]. The present review extends this literature with a study-level *N* = 270 audit linking primary disease area, clinical function, reported performance, dataset provenance, validation type, direct clinical comparison, implementation-oriented outcomes, and explainable artificial intelligence (XAI).

This review was designed to evaluate AI across the ophthalmic clinical pathway rather than as a diagnostic-accuracy review alone. Contemporary ophthalmic AI now encompasses screening, disease classification, prognostic prediction, treatment-response estimation, longitudinal monitoring, triage/referral, workflow implementation, and clinician-facing decision support [5,6,7,8,9]. Integrating these functions in one systematic review permits assessment of how AI is moving from image interpretation toward clinical management while preserving disease-, modality-, and task-specific distinctions.

Pediatric and pediatric-relevant applications were analyzed as a predefined subgroup within the broader evidence base. The primary objective was to characterize contemporary AI across screening, diagnosis/classification, prognosis, treatment-response prediction, monitoring, triage/referral, and clinical decision support, and to evaluate how disease area, model family, imaging/input modality, dataset characteristics, external validation, direct clinical comparators, advanced metrics, and explainability shape clinical readiness across ophthalmic eye care. The deliberately broad scope was chosen to evaluate AI across the clinical pathway within one eligibility-driven systematic framework, rather than to provide a topic-mapping or scoping inventory. Explicit optometry/optometrist involvement was audited separately rather than treated as a defining characteristic of the corpus.

## 2. Materials and Methods

### 2.1. Study Design and Reporting Standards

This study was conducted as a systematic review of AI applications in eye disease and ophthalmic care and was prepared according to the PRISMA 2020 statement [13]. The review was registered in PROSPERO under registration number CRD420251241901. The PRISMA flow diagram is included in the main text, and the completed PRISMA 2020 checklist is provided as Supplementary File S1.

Generative AI was not used for study design, eligibility decisions, study selection, data extraction, methodological appraisal, quantitative analysis, interpretation, or reference selection. During manuscript preparation, ChatGPT (OpenAI; https://chatgpt.com/) was used to assist with language refinement, organization, and editorial revision of author-generated text; all outputs were reviewed and verified by the authors.

### 2.2. Search Strategy and Information Sources

The search strategy was designed to capture AI applications relevant to ophthalmic diagnosis, screening, classification, prognosis, treatment-response prediction, monitoring, and clinical decision support. The declared information sources were PubMed/MEDLINE, Embase, Scopus, Web of Science, the Cochrane Library/CENTRAL, IEEE Xplore, and supplementary identification routes. The historical identification ledger documents 13,806 records and now reconciles exactly source by source. Six database sources contributed 13,789 records: PubMed/MEDLINE 3455; Embase 2290; Scopus 1671; Web of Science 1000; Cochrane Library/CENTRAL 800; and IEEE Xplore 4573. Supplementary identification contributed 17 records: Google Scholar 10 and citation/reference-list/manual searching 7. These source-level counts are components of, rather than additions to, the 13,806-record denominator. Source-specific strategies, retained execution/import documentation, and any remaining execution-history limitations are provided in Supplementary File S2.

The final publication-eligibility window was 1 January 2015 through 10 August 2026. Importantly, 10 August 2026 was the locked publication cutoff, not a synchronized execution date for every database. The original PROSPERO record stated no date restriction; Version 2.0, published 25 August 2026, aligned the public record with the operational eligibility window documented in the review materials. Full source-specific documentation and archive limitations are provided in Supplementary File S2.

Supplementary identification within the historical 13,806-record ledger comprised Google Scholar (*n* = 10) and citation/reference-list/manual searching (*n* = 7). Publisher-direct/current-awareness routes and iterative completeness checks were additionally used during later update/retrieval work; these were documented at report/study level where provenance was retained and were not added as unsupported new raw hits to the locked identification denominator. The formal post-November update log contained 20 candidate reports and 11 eligible original-study additions with retained study-level source routes. Before final synthesis, the screening dataset was closed against the locked publication-eligibility window of 10 August 2026 and the final PRISMA counts were consolidated within the same screening universe. The resulting flow comprised 12,762 records screened, 12,273 excluded at title/abstract screening, 489 reports sought for retrieval, 3 reports not retrieved, 486 reports assessed for eligibility, 216 full-text exclusions, and 270 included studies. Detailed source-provenance and screening-closure documentation are provided in Supplementary Data S1 and Supplementary File S2.

### 2.3. Eligibility Criteria

Eligible studies were original research evaluations of AI in an ophthalmic or eye-care context. The AI system was required to address at least one clinically relevant function: screening, diagnosis or disease classification, risk stratification, prognosis, treatment planning or treatment-response prediction, longitudinal monitoring, triage/referral, implementation, or clinician-facing decision support. Studies were eligible across adult and pediatric populations. Inherited retinal diseases, including retinitis pigmentosa, Stargardt disease, and related disorders, were explicitly eligible when they met the same clinical-function criteria. Purely technical measurement or segmentation without an eligible clinical function, synthetic-image generation/realism alone, and operational service prediction without an ophthalmic clinical function were excluded. Review articles, scoping reviews, viewpoints, and perspective papers were not included in original-study extraction or appraisal, although selected review-type publications were used for contextual discussion. A direct comparator was not required for eligibility; for synthesis, a direct clinical comparator was defined strictly as a human clinician/reader, conventional non-AI clinical method or score, or standard-care workflow directly compared on a relevant outcome. Expert or reference-standard ground truth alone was not classified as a direct clinical comparator. Clinician labels used solely as reference ground truth, AI-versus-AI comparisons, single-arm validation, and comparisons against a reference standard without an independently evaluated clinical comparator were likewise not classified as direct clinical comparators.

### 2.4. Study Selection and Data Extraction

Study selection was managed using Covidence (Veritas Health Innovation, Melbourne, Australia; https://www.covidence.org/) and predefined eligibility criteria. Titles and abstracts were screened first, followed by full-text review of potentially relevant reports. Because abstracts did not always provide enough detail about the AI application, clinical context, or outcome type, final eligibility was determined after full-text assessment when the article was available. Two reviewers independently screened titles/abstracts and full texts. Discrepancies were resolved by discussion and, when required, consultation with a third reviewer. In the retained paired final-study eligibility decision set, the two reviewers agreed on 260 of 270 decisions (96.3% observed agreement); the 10 discordant decisions were resolved by the third reviewer. This audit is limited to the retained final *N* = 270 study-level eligibility set and is not presented as agreement across all 12,762 title/abstract screening records. Reasons for full-text exclusion were documented and are provided in the Supplementary Materials.

Data extraction was performed independently by two reviewers using a structured, wide-format workbook. Discrepancies were resolved by discussion and, when required, consultation with a third reviewer. Complete study-level concordance was observed for 265 of 270 studies (98.1%); five studies contained a total of eight field-level discrepancies, primarily transcription or classification differences, all of which were resolved by source verification and reviewer consensus. Extracted fields included study identification, publication year, country/region where directly verifiable, study design, population or dataset, clinical indication, clinical function, AI model family, named architecture where verifiable, input modality, comparator or reference standard, validation approach, outcomes, exact performance metrics, key findings, limitations, and DOI or stable identifier. The verified extraction also included dataset source type, center structure, external-validation category, advanced performance metrics, and explainability/XAI. External validation was classified as clinical/geographic/prospective/real-world when evaluation used an independent clinical population, site, geography, prospective cohort, or routine-care workflow distinct from the development data. Technical external validation referred to independent public cross-dataset or device/domain evaluation without prospective or real-world clinical testing. Studies demonstrating both were coded as mixed validation. When independent external validation was reported but the retained record did not support a defensible subtype assignment, it was classified as externally validated–subtype unspecified. Internal split-sample, cross-validation, or held-out testing from the same source population was classified as internal/no independent external validation. Clinical and technical external validation were not treated as equivalent evidence of clinical transferability. For inclusive reporting, mixed studies were also counted as having a clinical component; therefore, 95/270 studies (35.2%) had any clinical/geographic/prospective/real-world external-validation component, comprising 86 clinical-only studies and 9 mixed clinical + technical studies. XAI coding classified Grad-CAM/CAM, XRAI, Integrated Gradients, saliency, and related heat-map methods as post hoc visual attribution; SHAP and related methods as feature attribution; and attention/interactive or intrinsically interpretable approaches in their respective XAI categories when explicitly presented as explainability; native segmentation/localization alone and LLM-generated narrative explanation alone were not counted as validated XAI.

All extracted variables were manually checked against the available primary-source record before synthesis. For mutually exclusive disease-level summaries, each study was assigned exactly one primary clinical area according to its dominant disease/clinical target; model family, including general-purpose LLM/VLM use, was treated separately as a cross-cutting characteristic rather than as a clinical-area category. For subgroup coding, a study was classified as pediatric/pediatric-relevant only when the report explicitly involved children, adolescents, infants/neonates, prematurity, or an intrinsically pediatric condition such as retinopathy of prematurity. Explicit optometry/optometrist involvement was counted only when an optometrist, optometric service, or optometry role was explicitly reported in the study record; author affiliation alone was not used to infer involvement, and this variable was not equated with a dedicated optometric setting. Candidate reports from the complete consolidated search period were adjudicated using the same final eligibility rule, and the comprehensive study-level evidence file is provided as Supplementary Data S1. Stage-specific reviewer-agreement audits are provided in Supplementary Data S1 and Supplementary File S3.

### 2.5. Methodological Appraisal

Methodological appraisal was performed using JBI critical appraisal tools matched as closely as possible to each study design [14]. Because the final evidence base contained diagnostic-accuracy/model-validation, cohort/prognostic/prediction, cross-sectional/content-evaluation, quasi-experimental/implementation, and randomized/interventional designs, no single checklist was appropriate across all studies. A design-specific JBI/adapted-JBI framework was therefore applied at item level to all 270 included studies. ‘Adapted’ denotes transparent mapping of the most applicable JBI design domains to the heterogeneous AI-study families; it does not denote creation of a new numerical quality instrument or a validated replacement for QUADAS-2, RoB 2, or ROBINS-I. Judgments were recorded as Yes, No, Unclear, or N/A; no author-created global lower/some/high-concern categories are used in the final synthesis, and no study was excluded solely because of appraisal results. The framework was piloted on the initial 55-study set to standardize operational interpretation and then applied across the final corpus. Two reviewers independently completed the final design-specific item-level appraisal across all 270 included studies. Across 2717 applicable paired item-level judgments (Yes/No/Unclear; design-inapplicable N/A pairs excluded), 2711 were concordant (99.8% observed agreement; Cohen’s kappa = 0.995); the six disagreements were resolved through reviewer consensus/final adjudication. N/A denotes an item that was not applicable to the study design, not unavailable information. The complete mapping rules, final item-level matrix, and appraisal-agreement audit are provided in Supplementary Table S1 and Supplementary File S3. Because the 200 diagnostic/model-validation reports included both classic diagnostic-accuracy evaluations and AI model-development/validation studies, a single whole-corpus QUADAS-2 structure was not uniformly applicable; similarly, RoB 2 and ROBINS-I are design-specific rather than whole-corpus solutions. The final report therefore does not claim equivalence among these instruments. Applying several parallel risk-of-bias instruments would fragment the whole-corpus appraisal into subset-specific outputs that assess different constructs, while many AI model-development/validation reports do not meet the classic index-test/reference-standard assumptions of QUADAS-2. We therefore retained one auditable design-mapped item-level appraisal layer across the complete heterogeneous corpus and report the protocol deviation and tool-specific limitations explicitly. Supplementary Table S1 now includes a design-to-domain Tool_Rationale_Crosswalk that maps each final design family to the item-level domains used and explicitly identifies the corresponding tool-specific limitations.

### 2.6. Evidence Synthesis and Protocol Updates

A meta-analysis was not performed because even large disease-specific subsets remained heterogeneous in clinical task, AI architecture, input modality, population, reference standard, validation design, thresholds, and reported performance metrics. A narrative synthesis was therefore used, combining study-level descriptive coding with disease × model, disease × modality, validation, comparator, advanced-metric, and XAI summaries. Despite its broad cross-disease and cross-function scope, this review was conducted as an eligibility-driven systematic review rather than a scoping or mapping review: all records were screened against predefined eligibility criteria, all included studies underwent structured study-level extraction and critical appraisal, and synthesis used predefined clinical and methodological variables. The descriptive synthesis was therefore intended to evaluate the distribution, strength, validation, comparators, and clinical-readiness characteristics of the eligible evidence base rather than merely catalogue topics. Counts were interpreted as evidence density and not as proof of model or modality superiority across heterogeneous tasks.

No formal assessment of reporting bias due to missing results was undertaken because no meta-analysis was performed and the evidence was synthesized narratively across heterogeneous designs and outcomes. No formal GRADE or other certainty-of-evidence rating was applied; confidence and transferability were interpreted qualitatively using item-level critical appraisal, validation type, comparator status, dataset provenance, and reporting limitations.

Two post-registration methodological updates were documented transparently. First, the eligible publication window was specified as 1 January 2015 through 10 August 2026, whereas the original PROSPERO registration stated no date restriction; the rationale was to focus on the contemporary clinical-AI era with the 1 January 2015 lower boundary preceding the major acceleration of deep-learning ophthalmic studies from 2016 onward [1,3]. Second, design-specific JBI appraisal was used instead of the initially listed QUADAS-2, RoB 2, and ROBINS-I framework because the final corpus comprised heterogeneous diagnostic, prognostic, cross-sectional, interventional, and implementation designs. Quantitative pooling was not undertaken because the protocol-specified condition of sufficient clinical and methodological homogeneity was not met. These updates did not change the core clinical review question and are documented in Supplementary File S2.

## 3. Results

### 3.1. Study Selection

Records identified across PubMed/MEDLINE, Embase, Scopus, Web of Science, the Cochrane Library, IEEE Xplore, and supplementary sources were considered within one consolidated review period from 1 January 2015 through 10 August 2026 and screened using the same eligibility criteria.

**Figure 1:**
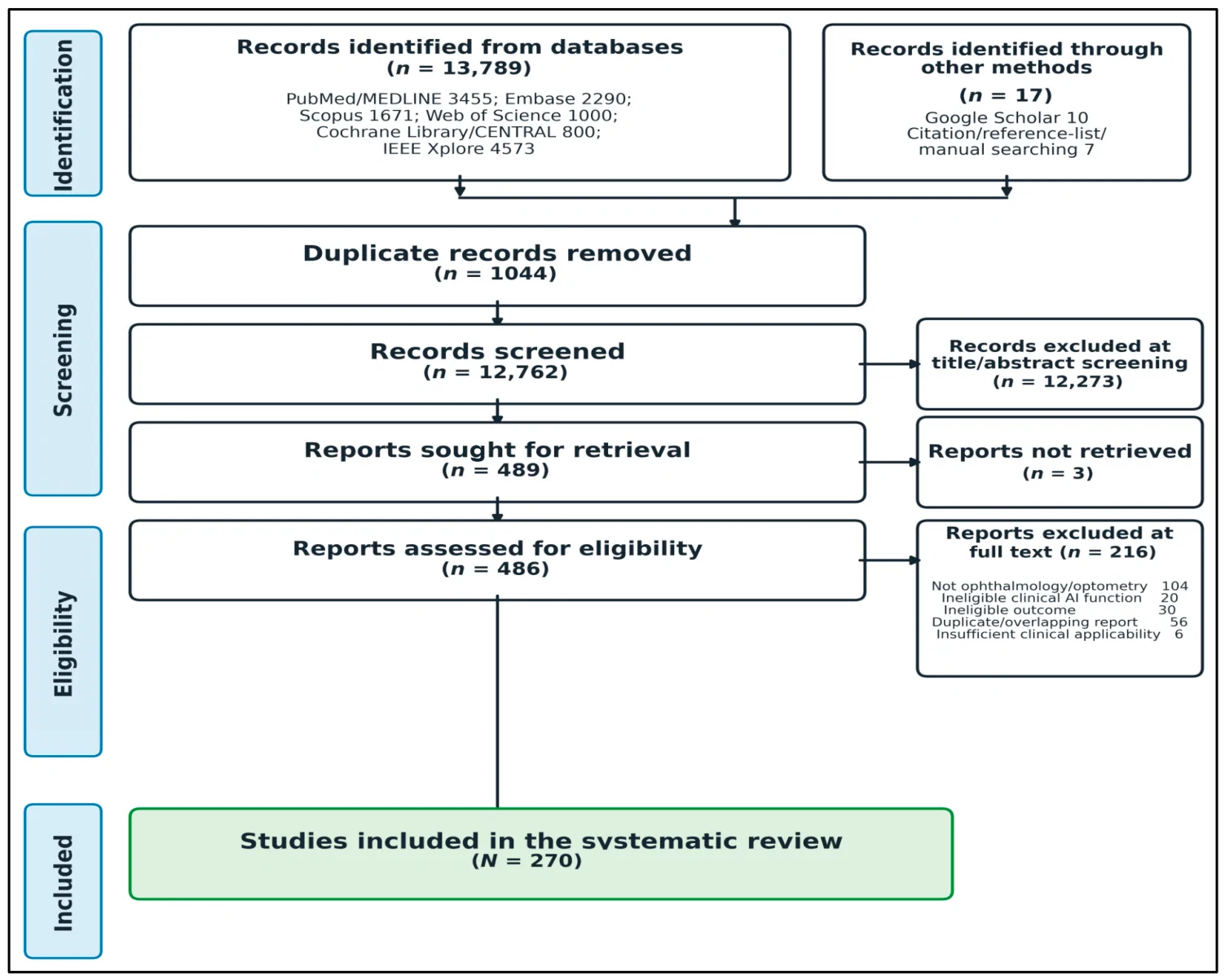
Preferred Reporting Items for Systematic Reviews and Meta-Analyses (PRISMA) 2020 flow diagram of study selection. Identification is displayed in two PRISMA streams: databases (*n* = 13,789: PubMed/MEDLINE 3455; Embase 2290; Scopus 1671; Web of Science 1000; Cochrane Library/CENTRAL 800; IEEE Xplore 4573) and other methods (*n* = 17: Google Scholar 10; citation/reference-list/manual searching 7). Together these streams comprise the historical 13,806-record denominator before deduplication. Detailed search provenance and later update/completeness documentation are provided in Supplementary Data S1 and Supplementary File S2. The two identification streams converge before deduplication; all downstream screening counts were rechecked against the final screening-closure audit in Supplementary Data S1 and Supplementary File S2.

The study-selection pathway is summarized in Figure 1 as a single PRISMA 2020 flow with separate identification streams for databases and other methods. Of 13,806 identified records (13,789 database records and 17 records identified through other methods), 1044 duplicates were removed and 12,762 records were screened. A total of 12,273 records were excluded at title/abstract screening, leaving 489 reports sought for retrieval. Three reports were not retrieved, and 486 reports were assessed for full-text eligibility. Of these, 216 were excluded, yielding a final evidence base of 270 included studies.

The 216 full-text exclusions comprised reports that were not ophthalmology/optometry (*n* = 104), had an ineligible clinical AI function (*n* = 20), had an ineligible outcome (*n* = 30), were duplicate/overlapping reports (*n* = 56), or had insufficient clinical applicability (*n* = 6). The retained historical full-text exclusion log combined duplicate publications and overlapping reports within this category; because a defensible record-level split was not preserved, separate retrospective counts were not reconstructed. The three reports that remained not retrieved were excluded from extraction and appraisal. Detailed search-source provenance and the audit trail used to finalize these unified counts are provided in Supplementary Data S1 and Supplementary File S2.

### 3.2. Characteristics of Included Studies

**Figure 2:**
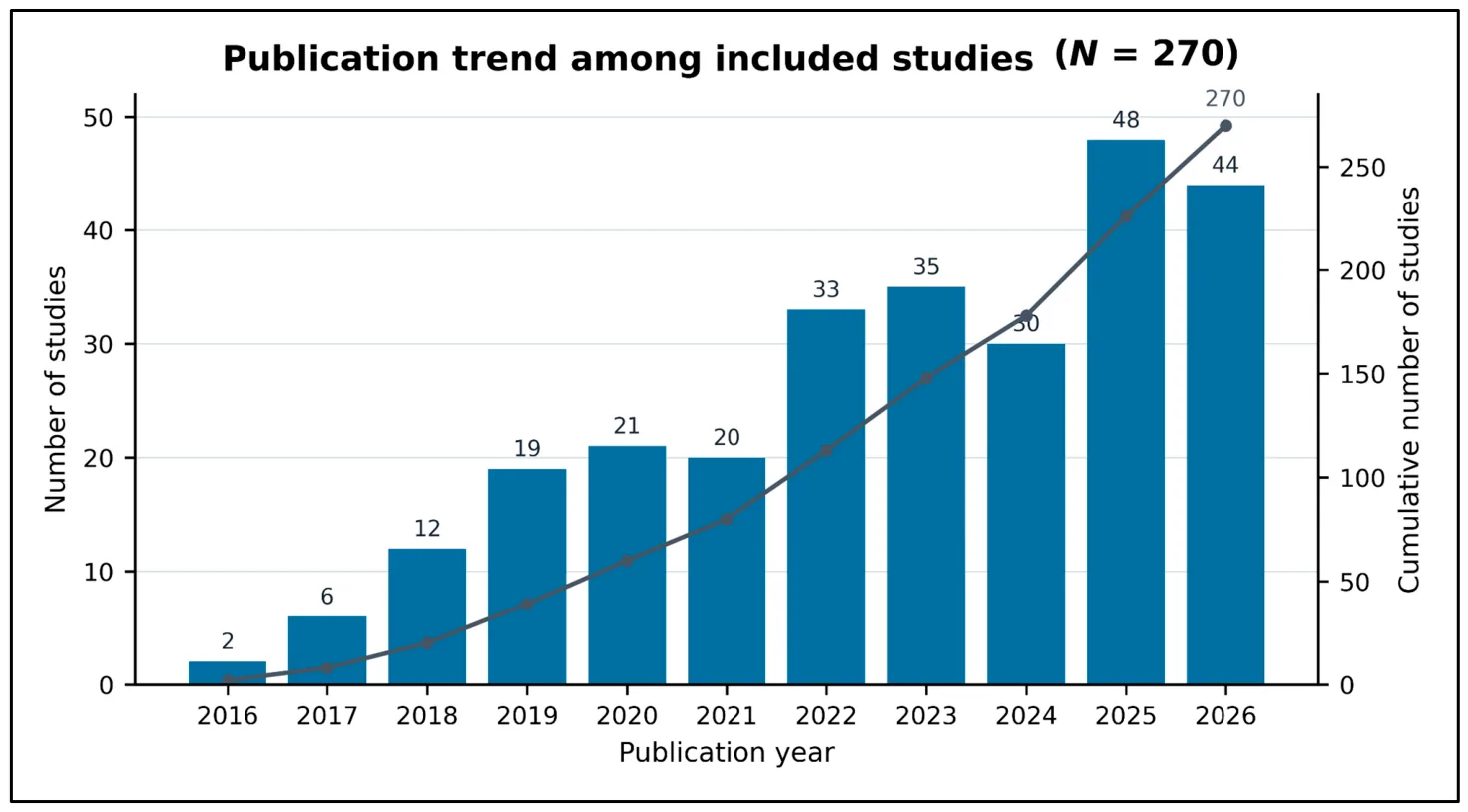
Publication trend among the 270 included studies. Bars show annual study counts, and the line shows the cumulative total. The 2026 count is partial through 10 August 2026.

The final review included 270 original studies published between 2016 and 2026. The evidence base was concentrated in recent years, with 190/270 studies (70.4%) published from 2022 through 2026 and 92/270 (34.1%) published in 2025–2026. Figure 2 summarizes the publication-year trend. The 2026 count is partial through the locked publication-eligibility cutoff of 10 August 2026.

Complete study-level bibliographic traceability for all 270 eligible reports is provided in Supplementary Data S1. Every included study is represented in the main reference list and mapped study-by-study in Supplementary Data S1 [3,15,16,17,18,19,20,21,22,23,24,25,26,27,28,29,30,31,32,33,34,35,36,37,38,39,40,41,42,43,44,45,46,47,48,49,50,51,52,53,54,55,56,57,58,59,60,61,62,63,64,65,66,67,68,69,70,71,72,73,74,75,76,77,78,79,80,81,82,83,84,85,86,87,88,89,90,91,92,93,94,95,96,97,98,99,100,101,102,103,104,105,106,107,108,109,110,111,112,113,114,115,116,117,118,119,120,121,122,123,124,125,126,127,128,129,130,131,132,133,134,135,136,137,138,139,140,141,142,143,144,145,146,147,148,149,150,151,152,153,154,155,156,157,158,159,160,161,162,163,164,165,166,167,168,169,170,171,172,173,174,175,176,177,178,179,180,181,182,183,184,185,186,187,188,189,190,191,192,193,194,195,196,197,198,199,200,201,202,203,204,205,206,207,208,209,210,211,212,213,214,215,216,217,218,219,220,221,222,223,224,225,226,227,228,229,230,231,232,233,234,235,236,237,238,239,240,241,242,243,244,245,246,247,248,249,250,251,252,253,254,255,256,257,258,259,260,261,262,263,264,265,266,267,268,269,270,271,272,273,274,275,276,277,278,279,280,281,282,283], together with DOI/PMID/stable identifiers where available. References [266,267,268] are contextual review-type publications and are not counted as included original studies.

**Figure 3:**
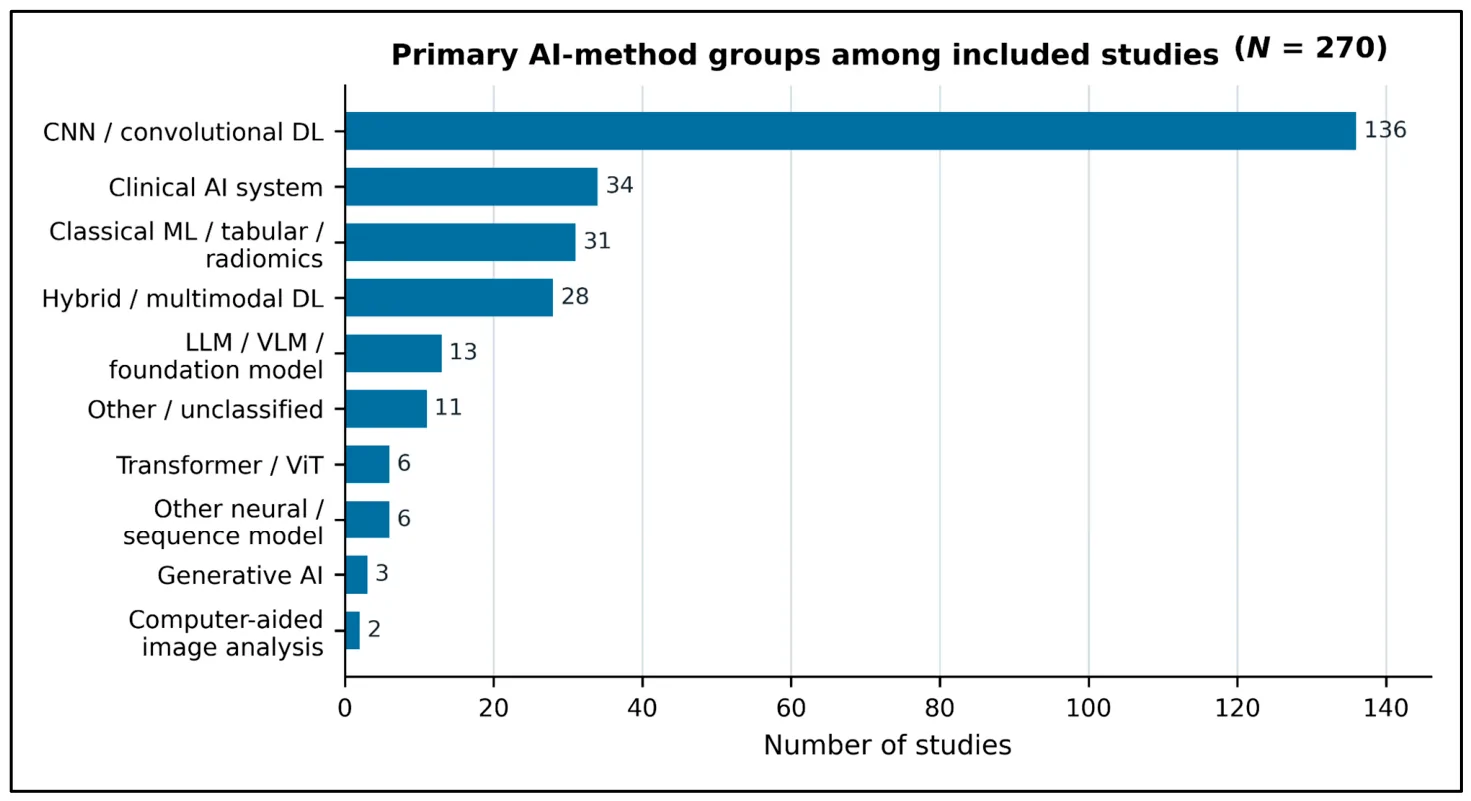
Primary AI-method groups in the final eligible evidence base (*N* = 270). Abbreviated labels are used for readability; counts describe evidence density and should not be interpreted as comparative superiority. AI, artificial intelligence; CNN, convolutional neural network; DL, deep learning; ML, machine learning; LLM, large language model; ViT, vision transformer; VLM, vision-language model.

The primary AI-method distribution is shown in Figure 3. CNN/convolutional deep learning remained the largest primary group (136/270), followed by clinical AI systems (34), classical ML/tabular/radiomics approaches (31), and hybrid/multimodal deep-learning systems (28). The included studies were also characterized according to major clinical area, direct-comparator status, external validation, and explainability (Table 1). Dataset source type, center structure, country/region verification, and recurrent named-dataset use are retained at study level in Supplementary Data S1 and were not inferred from author affiliation or current indexing. Country/region could be directly verified from study dataset/site/validation information for 103/270 studies (38.1%); for 167/270, a study country could not be assigned without inference from author affiliation. A uniform corpus-wide single- versus multicenter count is not reported where the retained study-level evidence cannot support a defensible aggregate. Explicit optometry/optometrist involvement was identified in only 3/270 studies (1.1%) under the strict study-level rule, indicating sparse explicit optometry representation in the evidence base.

A recurrent-dataset/cohort audit identified 45/270 studies that mentioned at least one of 10 recurrent named dataset/cohort families. This indicates potential data dependence or dataset reuse rather than proof that identical patient samples were analyzed, because public datasets may be used with different subsets, preprocessing, and endpoints. The detailed study IDs and overlap notes are provided in Supplementary Data S1.

### 3.3. Clinical Scope and AI System Characteristics

The evidence base covered a broad range of ophthalmic diagnostic and clinical-management applications. Disease-specific evidence density was greatest in diabetic retinopathy/DME (82 studies), retinal or multi-disease ophthalmic AI (52), glaucoma (46), and AMD/macular disease (29), followed by retinopathy of prematurity (15), inherited retinal disease (15), neuro-ophthalmology/optic-nerve applications (eight), refractive error/myopia (seven), and strabismus/amblyopia (seven). Cornea/ocular-surface/keratoconus and lens/cataract each contributed three studies, retinal detachment two, and ocular-surface neoplasia one. General-purpose LLM/VLM studies were assigned to these mutually exclusive primary clinical areas according to their dominant disease/clinical target and analyzed separately in a cross-cutting model-family audit. These counts describe evidence density rather than comparative clinical superiority.

Fundus/retinal photography remained the predominant input across the corpus, with OCT and multimodal imaging/clinical inputs also strongly represented. Because modality labels can overlap within multimodal studies, the final narrative emphasizes disease-specific patterns rather than treating modality totals as mutually exclusive. These disease × AI-method × modality × outcome patterns are summarized later in the Results, while the exact study-level coding is retained in Supplementary Data S1.

### 3.4. Reported Performance and Outcome Domains

Actual study-reported discrimination performance is summarized descriptively by primary clinical area in Table 2. Because thresholds, endpoints, reference standards, datasets, and validation designs were heterogeneous, values were not pooled or weighted; the table reports unweighted minimum–maximum ranges and the number of contributing studies for AUC/AUROC, sensitivity, and specificity. Outcome-domain coding was also retained at the study level for workflow/efficiency, reliability/agreement, usability/acceptability, trust, and safety/potential harm. All 13 mutually exclusive primary clinical areas are represented in Table 2; when a metric was not safely extractable from the retained study-level result, this is stated rather than imputed. The DR/DME lower-bound AUC of 0.380 was rechecked against the retained study-level extraction and source record and was confirmed rather than treated as a transcription error. It arose from Google Vertex AI for referable diabetic retinopathy in an independent institutional patient-level external test cohort within a six-platform AutoML comparison; other platforms in the same study achieved referable-DR AUCs of 0.93–0.94, indicating platform-specific poor/unstable discrimination [268].

**Figure 4:**
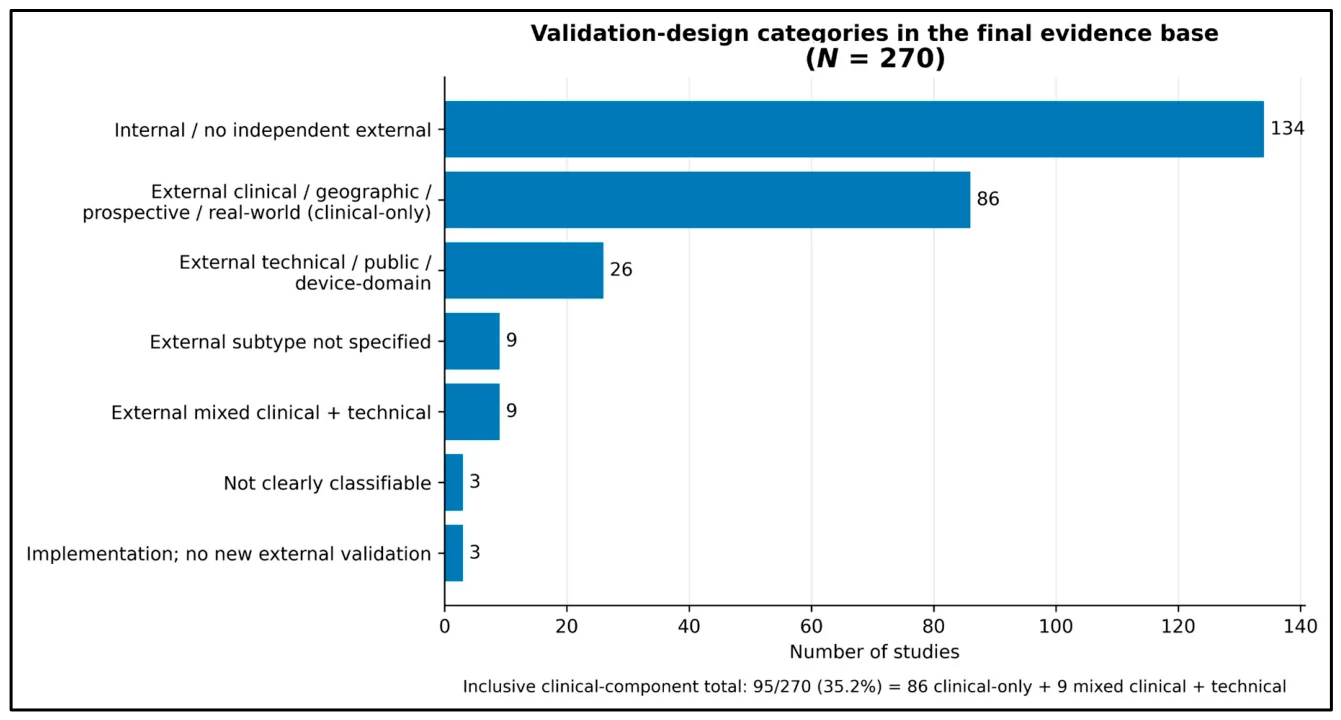
Validation-design categories in the final eligible evidence base (*N* = 270). Clinical/geographic/prospective/real-world external validation is distinguished from technical public cross-dataset or device/domain generalization and from external-validation records whose subtype could not be safely specified. The plotted validation categories are mutually exclusive: 86 studies were clinical-only and 9 were mixed clinical + technical; consequently, 95/270 studies (35.2%) had any clinical/geographic/prospective/real-world external-validation component.

Beyond discrimination metrics, reliability/agreement was explicitly identified in 44/270 studies, efficiency/workflow/implementation in 26/270, formal usability/acceptability/satisfaction in 1/270, and safety/potential harm in 2/270; no study met the strict coding rule for a formal trust/user-confidence outcome. Advanced metrics such as F1/F2, MCC, balanced accuracy, precision-recall/AUPRC, agreement measures, continuous prediction error, and segmentation/overlap metrics were retained at study level without pooling or imputation. Explicit or intrinsically interpretable XAI was identified in 90/270 studies (33.3%). Within the final coding framework, XAI approaches were categorized as post hoc visual attribution/saliency, feature attribution or intrinsic interpretability, attention/interactive explanation, or other explicit XAI/visualization approaches; native localization/segmentation without a separate explanation method and LLM-generated explanation text alone were not counted as validated XAI. Validation-design categories are summarized in Figure 4.

A focused general-purpose LLM/VLM audit identified 13 included studies evaluating ChatGPT/GPT, Gemini, Claude, Bing Copilot, or a ChatGPT-powered multimodal system; six included a strict direct clinical comparator. This cross-cutting model-family audit is independent of the 13 mutually exclusive primary clinical-area categories; each study remains assigned to its dominant disease/clinical target in Supplementary Data S1. Within-study comparisons were heterogeneous and were not pooled across prompts, versions, modalities, case mix, or reference standards. GPT-4 outperformed GPT-3.5 in neuro-ophthalmic case diagnosis (82% vs. 59%) but remained slightly below two neuro-ophthalmologists (86% each) [151]. In ophthalmic registration/diagnosis, GPT-4 improved on GPT-3.5 and approached resident performance [153]; GPT-4V remained below human readers on multimodal clinical cases [159]. Across 40 ophthalmic-emergency vignettes, ChatGPT-5, Gemini 2.5 Pro, and Claude Opus 4.1 produced similar overall scores with no statistically significant overall difference [167]. In ROP scenarios, ChatGPT achieved 90% correct responses versus 77.5% for fellowship trainees and 96% for ROP experts [242]. The complete focused comparison is provided in Supplementary Data S1, sheet ‘General_Purpose_LLM_Audit’.

Table 3 links the major clinical areas to their dominant AI-method/modality patterns and principal clinical outcome emphasis. Exact study-level performance metrics, advanced metrics, validation fields, and XAI coding are provided in Supplementary Data S1.

Reported performance metrics should not be interpreted as directly comparable across studies because datasets, validation methods, disease prevalence, imaging quality, thresholds, and reference standards varied substantially.

### 3.5. Methodological Appraisal Summary

The design-specific JBI/adapted-JBI appraisal of all 270 included studies mapped 200 reports to diagnostic-accuracy/model-validation domains, 48 to cohort/prognostic/prediction domains, 13 to cross-sectional/content-evaluation domains, five to quasi-experimental/implementation domains, and four to randomized/interventional domains. The final synthesis reports the item-level Yes/No/Unclear/N/A judgments directly and does not use an author-created global lower/some/high-concern score. Across 2717 applicable paired item-level judgments, reviewer agreement was 99.8% (2711 concordant; Cohen’s kappa = 0.995); six disagreements were resolved in the final consensus/adjudication. Recurrent limitations included selected or incompletely described sampling, incomplete reporting of exclusions or ungradable cases, unclear blinding/independence of reference assessment in some retrospective studies, limited independent external validation, and incomplete reporting of confounding, follow-up, or calibration where applicable.

No study was excluded solely on the basis of JBI appraisal. Instead, item-level appraisal was used to contextualize the strength and transferability of the evidence. The complete design-mapped Yes/No/Unclear/N/A matrix for all 270 included studies is presented in Supplementary Table S1.

### 3.6. Clinical Functions Across Diagnosis and Management

Overall, the evidence base reflects a transition from narrow diagnostic classification toward broader clinical-management functions. Across the 270-study corpus, screening/detection/triage was coded in 145 studies, diagnosis/classification/grading in 132, prognosis/risk prediction in 37, treatment planning/response in 14, monitoring/longitudinal follow-up in 15, decision support/management in 24, and workflow/implementation/access in 26. Because functions were non-mutually exclusive, 94 unique studies had at least one management-oriented code. The year-by-year matrix is shown in Table 4 and retained in full in Supplementary Data S1.

Task-specific performance varied markedly across diseases, endpoints and validation settings. Several image-based applications reported high discrimination, but the broad ranges in Table 2 and the heterogeneity of reference standards and datasets preclude a single summary estimate. Newer studies increasingly included external validation, multicenter datasets, real-world deployment, and clinician-facing decision support, yet safety, equity, implementation feasibility, patient-centered outcomes, calibration, and performance drift remained inconsistently evaluated. Inherited retinal disease was represented by 15 eligible studies, including a multicenter randomized clinician-assistance evaluation; emerging transformer, multimodal, foundation-model, and vision-language applications were interpreted cautiously because external clinical validation and failure-mode reporting remain variable.

Importantly for clinical management, the corpus included AI applications for anti-VEGF response prediction, retreatment decisions, progression and recurrence risk, longitudinal disease or biomarker monitoring, referral/triage, workflow integration, and clinician-facing decision support. Zhao et al. [17], for example, evaluated multimodal prediction of visual and anatomical response to anti-VEGF therapy in neovascular AMD. The final evidence base also included a randomized clinician-assistance trial for inherited retinal disease [280], a prospective/noninferiority AI-OCT pathway for diabetic macular oedema [271], and real-world autonomous diabetic-retinopathy screening integration [282]. These systems should be interpreted as decision-support or workflow tools rather than autonomous therapeutic systems; prospective comparative evidence remains necessary before AI-guided treatment decisions can be considered routine care.

## 4. Discussion

### 4.1. Principal Findings

This systematic review synthesizes 270 original studies evaluating AI across ophthalmic screening, diagnosis/classification, prognosis, treatment-response prediction, monitoring, and clinical decision support. The evidence base confirms rapid growth in image-rich ophthalmic AI while showing uneven evidence density across diseases, model families, modalities, validation designs, and clinical-translation outcomes. Importantly, clinically oriented AI is no longer confined to diagnosis: treatment-response, progression, triage, workflow, and management-support applications are increasingly represented.

The review also highlights an important gap between technical performance and clinical translation. Although many studies reported high diagnostic or prognostic performance, fewer consistently evaluated safety, reproducibility, patient-centered outcomes, equity, workflow impact, or longitudinal effectiveness. These gaps limit immediate generalization and indicate that AI should be interpreted as a supervised diagnostic and decision-support adjunct rather than an autonomous replacement for clinical expertise.

### 4.2. Relationship to Recent Review-Type Literature

Prior ophthalmic-AI reviews establish the breadth and importance of the field but differ from the present analysis. Srivastava et al. provided a broad review of AI/ML applications across ophthalmology [10]; Costin et al. synthesized advantages and limitations [11]; and Savastano et al. emphasized progress, challenges, ethics, bias, privacy, explainability, and regulation [12]. More focused 2026 reviews address disease-specific diagnostic accuracy, AI-enabled devices, clinical integration, and service-delivery pathways [5,6,7,8,9]. The present review adds a structured *N* = 270 study-level dataset and a quantitative cross-pathway audit of disease assignment, unpooled performance, function-by-year trends, validation type, direct clinical comparators, recurrent dataset use, pediatric/optometry scope, and XAI.

Recent review-type literature was used to contextualize the present findings within broader developments in ophthalmic AI, diagnostic translation, and care-pathway implementation (Table 5).

### 4.3. From Diagnostic Performance to Clinical Management and Implementation

The findings are relevant to both ophthalmic diagnosis and clinical management because included systems addressed disease detection, grading and classification, prognostic prediction, treatment-response estimation, monitoring, triage, and decision support. Diagnostic applications are clinically attractive in screening programs, primary/community eye-care pathways, resource-limited settings, and subspecialty clinics where imaging volume is high. Management-oriented applications may additionally support treatment selection, retreatment timing, disease surveillance, and referral prioritization. Across these use cases, implementation should remain context-specific: AI systems should be externally validated in the intended population and workflow, monitored for performance drift, and integrated with clinician oversight, referral pathways, and quality-assurance mechanisms. Real-world and pragmatic implementation studies, including non-ophthalmologist-led diabetic-retinopathy screening and routine clinical deployment, further illustrate the importance of workflow-specific validation [21,22].

For clinical interpretation, AUROC summarizes discrimination across decision thresholds but does not indicate the consequences of a specific operating threshold. Sensitivity reflects the ability to identify affected patients and is therefore particularly relevant when missed disease carries substantial harm, whereas specificity reflects the ability to avoid false-positive classification and unnecessary referral or testing. Predictive values are additionally influenced by disease prevalence; consequently, high discrimination metrics should be interpreted together with threshold choice, calibration, false-positive and false-negative consequences, and the intended clinical pathway.

For AI to produce clinical benefit, future evaluations should report reference standards, image quality procedures, population characteristics, disease spectrum, external validation, subgroup performance, calibration, false-positive and false-negative consequences, and downstream workflow effects. Treatment- and management-related systems should additionally report clinically meaningful therapeutic endpoints, decision thresholds, treatment or monitoring consequences, and whether AI changes outcomes compared with usual care. High discrimination alone does not establish that an AI system improves access, reduces avoidable delays, supports safe non-ophthalmologist-led screening, optimizes treatment, or improves patient outcomes.

Deployment generalizability may also be affected by recruitment and sampling, retrospective versus prospective data collection, imaging device/manufacturer and acquisition protocol, and demographic composition; race/ethnicity was not reported consistently enough across the corpus to support a uniform subgroup analysis.

Disease-, model-, and modality-specific patterns provide additional context for clinical translation. DR/DME (82 studies), retinal/multi-disease AI (52), glaucoma (46), and AMD/macular disease (29) were the most densely studied areas, whereas corneal/ocular-surface disease, lens/cataract applications, ocular neoplasia, retinal detachment, and several pediatric or rare-disease tasks were comparatively sparse. Confirmed external validation was identified in 130/270 studies (48.1%), including 95/270 (35.2%) with any clinical, geographic, prospective, or real-world external-validation component (86 clinical-only and nine mixed clinical + technical); 49/270 (18.1%) had a strict direct clinical comparator. These categories are not equivalent in evidentiary strength, and study volume should be interpreted as evidence density rather than proof of clinical maturity.

From a therapeutic-management perspective, the most directly relevant applications were those predicting anti-VEGF response, retreatment need, disease progression or recurrence, and longitudinal biomarker change. Such tools could support individualized surveillance intervals and treatment planning, including multidisciplinary medication-management decisions, but the evidence is predominantly predictive rather than interventional. Accordingly, these systems should support—not replace—ophthalmologist judgment and should not be interpreted as autonomous prescribing or dosing tools. Treatment-related examples include AI-supported prediction of outcomes after intravitreal dexamethasone and anti-VEGF therapy and prediction of recurrence risk [17,18,19].

Explainability and emerging foundation-model approaches also require method-specific interpretation. Grad-CAM/CAM, XRAI, Integrated Gradients, saliency, and related visual-attribution methods can indicate image regions associated with a prediction but do not establish causal reasoning or explanation fidelity. SHAP and related feature-attribution methods can quantify feature contributions in structured or multimodal models but remain sensitive to feature dependence and background distributions. Attention maps should not automatically be equated with explanation. Foundation, vision-language, and multimodal models offer potential for integrating images, text, and clinical context, but current evidence remains sensitive to prompt/task definition, dataset composition, domain shift, and model version; prospective ophthalmic validation and transparent failure analysis are therefore needed before routine-deployment claims.

### 4.4. Pediatric and Inherited-Retinal-Disease Evidence

Pediatric and pediatric-relevant evidence included 30/270 studies (11.1%) spanning retinopathy of prematurity, childhood myopia and refractive-risk prediction, amblyopia and strabismus, pediatric optic-disc assessment, and other child-relevant eye-care tasks. In parallel, the targeted IRD reassessment retained 15 clinically eligible studies, including retinitis pigmentosa, Stargardt disease, broader inherited-retinal-disease classification, genotype/phenotype prediction, visual-function estimation, and the randomized clinician-assistance evaluation included in the final evidence base [280]. The subgroup was not broadened selectively: technical-only segmentation or measurement studies without an eligible clinical function were excluded under the same rule used for all disease areas. Explicit optometry/optometrist involvement was identified in only 3/270 studies (1.1%); accordingly, the review does not interpret the corpus as broadly representative of dedicated optometric settings or optometric practice.

Pediatric implementation also requires attention to age-specific disease spectrum, ocular development, cooperation during imaging, and referral thresholds. Models trained predominantly on adult or clinic-based datasets may not generalize well to children, particularly when image quality is affected by fixation, cooperation, or limited acquisition windows. Because delayed detection or referral in childhood may have long-term visual and developmental consequences, future pediatric evaluations should report age distribution, disease stage, imaging feasibility, false-negative consequences, and whether AI support improves timely referral or monitoring.

### 4.5. Strengths and Limitations

Strengths of this review include PROSPERO registration, PRISMA-oriented reporting, a defined contemporary publication-eligibility window from 1 January 2015 through 10 August 2026, structured study-level documentation for 270 eligible reports, independent duplicate data extraction, design-specific JBI/adapted-JBI item-level appraisal of all included studies, and direct bibliographic traceability between the manuscript and Supplementary Data S1. Final screening closure applied the locked 10 August 2026 publication cutoff consistently across the retained screening dataset before synthesis.

The main limitations arise from the included evidence base and the retained search/archive documentation. Studies were heterogeneous in clinical indication, AI architecture, input modality, comparator, reference standard, validation method, outcome definition, and reporting completeness; this limited direct comparability and made meta-analysis inappropriate. The final publication-eligibility cutoff was 10 August 2026, but this was not a synchronized execution date for every database, and a verbatim final native rerun/export is not retained for every source. Three potentially eligible reports (F001, F004, and F014) remained not retrieved and were not extracted or appraised. F014 is retained as a supplementary AMEDEO/British Journal of Ophthalmology current-awareness route rather than being retrospectively relabeled as a database record based on current indexing. The retained screening/eligibility agreement audit covers the final *N* = 270 study-level eligibility decision set and should not be interpreted as an agreement estimate across all 12,762 title/abstract screening records. The review was limited to English-language publications, and 2026 evidence is necessarily partial. Although all 270 included studies underwent item-level appraisal, source depth was not identical for every report, and Unclear judgments were retained when the verified primary source did not support a confident determination. Because the review deliberately retained one design-mapped JBI/adapted-JBI appraisal layer rather than parallel instrument-specific re-scoring, the findings are reported as transparent critical-appraisal judgments rather than formal QUADAS-2, RoB 2, or ROBINS-I risk-of-bias ratings. This preserves a consistent whole-corpus appraisal across heterogeneous designs while avoiding any claim that the instruments are interchangeable.

### 4.6. Future Research Directions

Future studies should prioritize prospective and externally validated designs, transparent reporting of datasets and algorithms, standardized diagnostic and AI performance metrics, calibration, clinically meaningful comparators, subgroup and equity analyses, safety monitoring, and real-world implementation outcomes. For clinical translation, studies should move beyond accuracy alone and evaluate whether AI improves triage, reduces workload, increases access, prevents avoidable vision loss, supports shared decision-making, improves treatment selection or monitoring, or changes patient outcomes in routine eye-care pathways. Densely studied areas now need multicenter clinical validation, direct comparators, failure/ungradable-case reporting, calibration, and downstream patient or workflow outcomes, whereas sparser corneal, cataract/lens, ocular-neoplasia, retinal-detachment, pediatric, and rare-disease applications need larger disease-specific cohorts. Treatment-response and management-oriented AI should be tested against usual-care decisions and clinically meaningful therapeutic endpoints. XAI studies should evaluate explanation fidelity and clinical usefulness, and foundation/vision-language studies should report locked model versions, prompts, safety/failure modes, and independent ophthalmic validation.

Future deployment studies should therefore report sampling and acquisition methods, device/manufacturer and imaging protocol, relevant demographic characteristics including race/ethnicity when available, and cross-device and subgroup validation and calibration.

## 5. Conclusions

This systematic review identified 270 original studies showing that ophthalmic AI increasingly spans the clinical continuum from screening and diagnosis to prognosis, treatment-response prediction, monitoring, triage, workflow integration, and decision support. The evidence base remains dominated by image-based deep learning and retinal imaging, while multimodal, transformer, foundation-model, and vision-language approaches are expanding the scope of AI-supported eye care. However, study volume is not synonymous with strong clinical evidence: translation remains limited by heterogeneous designs and outcomes, incomplete independent validation, few direct clinical comparators, and limited evaluation of calibration, safety, workflow effects, treatment consequences, equity, and patient outcomes. Only 95/270 studies (35.2%) included any clinical/geographic/prospective/real-world external-validation component, including nine studies with mixed clinical + technical validation. Future work should prioritize prospective, externally validated, clinically integrated systems that demonstrate measurable patient or pathway benefit.

## Figures and Tables

**Table 1:** Summary characteristics of the final 270-study evidence base. AMD, age-related macular degeneration; DME, diabetic macular oedema; XAI, explainable artificial intelligence.

Characteristic	*n* (%)	Interpretation
Included studies	270	Final eligible evidence base after final screening closure
Diabetic retinopathy/DME	82 (30.4%)	Largest disease-specific evidence area
Glaucoma	46 (17.0%)	Diagnostic, screening, progression and functional-prediction studies
Retinal/multi-disease ophthalmic AI	52 (19.3%)	Broad retinal, multi-disease and general ophthalmic systems
AMD/macular disease	29 (10.7%)	Includes progression and treatment-response applications
Retinopathy of prematurity	15 (5.6%)	Predominantly retinal-image screening/severity assessment
Inherited retinal disease	15 (5.6%)	Rare-disease, genotype/phenotype and clinician-support applications
Neuro-ophthalmology/optic nerve	8 (3.0%)	Non-glaucomatous optic-disc and neuro-ophthalmic applications
Direct clinical comparator	49 (18.1%)	Strict direct-comparator coding; reference ground truth alone not counted
Any confirmed external validation	130 (48.1%)	Clinical, technical, mixed, or external subtype retained after final coding audit
Any clinical/geographic/prospective/real-world external-validation component	95 (35.2%)	Inclusive total: 86 clinical-only + 9 mixed clinical + technical external-validation studies
Explicit/intrinsic XAI	90 (33.3%)	Excludes native segmentation alone and unsupported LLM explanation text

**Table 2:** Descriptive unweighted ranges of study-reported AUC/AUROC, sensitivity, and specificity by primary clinical area. Values are not pooled; *n* denotes the number of studies contributing the corresponding metric. AUC, area under the curve; AUROC, area under the receiver operating characteristic curve.

Clinical Area	*n*	AUC/AUROC *n*	AUC/AUROC Range	Sensitivity *n*	Sensitivity Range	Specificity *n*	Specificity Range
DR/DME	82	38	0.380–1.000	53	56.4–100.0%	52	40.7–100.0%
Glaucoma	46	20	0.696–0.996	19	43.0–100.0%	15	68.0–100.0%
Retinal/multi-disease	52	14	0.540–0.997	15	17.0–99.7%	15	61.7–99.9%
AMD/macular	29	7	0.720–0.990	4	84.6–98.9%	4	80.0–99.5%
ROP	15	5	0.900–0.993	8	70.6–100.0%	8	58.6–98.0%
IRD	15	4	0.741–0.993	4	88.3–99.8%	3	91.2–98.4%
Neuro-ophthalmology/optic nerve	8	5	0.880–0.995	4	75.6–98.0%	4	72.5–98.9%
Refractive error/myopia	7	3	0.941–0.990	1	87.8–98.6%	1	93.7–98.8%
Strabismus/amblyopia	7	4	0.908–0.998	4	74.0–99.1%	4	67.8–98.6%
Cornea/ocular surface/keratoconus	3	2	0.750–0.930	1	85.7%	1	97.8%
Lens/cataract	3	0	Not extractable	1	98.5%	1	91.6%
Retinal detachment	2	2	0.876–0.910	1	94.0%	1	80.0%
Ocular surface/neoplasia	1	1	0.894–0.998	0	Not extractable	0	Not extractable

**Table 3:** Disease × AI-method × modality × outcome synthesis in the final 270-study evidence base. Patterns describe evidence density and do not imply superiority across heterogeneous tasks.

Clinical Area (*n*)	AI-Method and Modality Pattern	Main Outcome Emphasis
DR/DME (82)	CNN and deployed clinical AI systems; fundus photography dominant, with OCT/multimodal pathways also represented	Screening/grading; DME detection; referral; real-world implementation
Glaucoma (46)	CNN, classical ML, multimodal and interpretable screening models; fundus, OCT and visual-field inputs	Detection; referral; incident-risk/progression prediction; VF/RNFL prediction
Retinal/multi-disease AI (52)	CNN, hybrid/multimodal, AutoML and general-purpose reasoning systems across fundus, OCT, text and multimodal clinical inputs	Multi-disease classification; diagnosis; triage; referral; management/decision support
AMD/macular disease (29)	CNN/hybrid models using OCT, fundus and multimodal inputs	Conversion/progression; geographic-atrophy growth; treatment-response prediction
ROP (15)	Predominantly CNN-based retinal-image systems	Plus/severity/stage classification; screening; treatment/referral prediction
IRD (15)	Multimodal, CNN and transformer/foundation approaches	IRD classification; genotype/phenotype prediction; clinician decision support
Neuro-ophthalmology/optic nerve (8)	Image-based deep learning with fundus/OCT inputs	Papilledema/optic-disc classification; visual-function/risk assessment
Refractive error/myopia (7)	Classical ML and deep/hybrid models using imaging and clinical variables	Myopia onset/progression; refraction prediction
Strabismus/amblyopia (7)	Classical ML, deep models and LLM/XAI approaches with eye-tracking/stereovision or image inputs	Screening/classification; alignment; amblyopia diagnosis/risk; explainable decision support
Cornea/ocular surface/keratoconus (3)	CNN/classical ML using anterior-segment/corneal information	Keratoconus and ocular-surface classification
Lens/cataract (3)	Image-based or clinical AI using slit-lamp/clinical inputs	Diagnosis, severity and treatment recommendation
Retinal detachment (2)	ML/CNN using registry or retinal-imaging inputs	Outcome prediction and retinal-detachment detection
Ocular surface/neoplasia (1)	CNN using ocular-surface/slit-lamp imaging	Multi-class ocular-surface tumor diagnosis

**Table 4:** Clinical-function counts by publication year. Clinical functions are non-mutually exclusive; “management-oriented” is a unique-study count with at least one management-oriented code.

Year	All	Screen/Triage	Diagnosis/Grading	Prognosis	Treatment	Monitoring	Decision Support	Workflow	Management-Oriented
2016	2	2	0	0	0	0	0	0	0
2017	6	4	4	1	1	0	2	0	2
2018	12	9	7	2	0	0	2	1	5
2019	19	12	10	3	1	1	5	0	8
2020	21	8	10	6	1	5	0	1	8
2021	20	13	11	1	0	0	0	1	2
2022	33	21	15	3	2	1	1	3	9
2023	35	17	18	2	1	1	2	1	7
2024	30	15	10	3	2	3	3	4	12
2025	48	19	24	8	5	3	4	4	17
2026	44	25	23	8	1	1	5	11	24
Total	270	145	132	37	14	15	24	26	94

**Table 5:** Contextual comparison with prior and recent ophthalmic-AI review literature.

Reference	Main Focus	Relevance to the Present Review
Srivastava et al., 2023 [10]	Broad AI/ML review across ophthalmology	Important field overview; the present review adds an auditable 270-study systematic dataset, performance ranges, function-year analysis, and validation/comparator/XAI coding.
Costin et al., 2025 [11]	Advantages and limitations of AI in ophthalmology	Narrative clinical/technical overview; the present review quantifies original-study evidence and implementation/validation gaps.
Savastano et al., 2025 [12]	Progress, challenges and ethical implications	Comprehensive challenges/ethics perspective; the present review adds study-level quantitative synthesis across clinical functions and validation tiers.
Chen et al., 2026 [5]	DR screening diagnostic accuracy/meta-analysis	Disease/task-specific quantitative synthesis; the present review is broader and does not pool non-comparable outcomes.
Kawamoto et al., 2026 [6]	Retinal detachment and ultra-widefield diagnostic accuracy	Focused imaging/disease review; the present review places such evidence within a cross-disease clinical pathway.
Grzybowski & Jin 2026 [7]	AI-based devices for DR screening	Device/regulatory focus; the present review audits validation type and direct-comparator evidence across diseases.
Gurnani & Kaur 2026 [8]	Clinical integration of ophthalmic AI	Clinical-integration perspective; the present review supplies study-level counts, performance, and reproducible categorization.
Poschkamp et al., 2026 [9]	AI and telemedicine pathways	Service-delivery and telemedicine perspective; the present review links implementation questions to the wider original-study evidence base.
Present review	270 original studies, 2016–10 August 2026	Systematic cross-pathway synthesis with corrected disease/design coding, unpooled performance ranges, function-year counts, validation tiers, comparator audit, dataset reuse, pediatric/optometry scope, and XAI taxonomy.

## Data Availability

All data generated or analyzed during this review are included in the article and its Supplementary Materials. Supplementary Data S1 provides the comprehensive study-level evidence table and manuscript-reference mapping for all 270 eligible reports, and Supplementary Table S1 provides the design-specific JBI/adapted-JBI item-level appraisal matrix.

## Supplementary Materials

The following supporting information can be downloaded at https://www.mdpi.com/article/10.3390/jcm15207957/s1, Supplementary Data S1: Comprehensive study-level evidence workbook for all 270 eligible studies, including manuscript-reference mapping, bibliographic identifiers, clinical area and function, AI model/model family, input modality, dataset/sample characteristics, validation, direct clinical comparator status, performance metrics, XAI, coding and audit sheets, screening/eligibility and data-extraction agreement audits, search chronology, formal-update source provenance, final screening-closure and provenance documentation, and a focused 13-study general-purpose LLM/VLM comparison audit. Supplementary Table S1: Design-specific JBI/adapted-JBI item-level critical appraisal matrix for all 270 included studies with Yes/No/Unclear/N/A judgments, design-mapping notes, an appraisal-agreement audit, and a design-to-domain Tool_Rationale_Crosswalk clarifying the relationship to QUADAS-2, RoB 2, and ROBINS-I without claiming tool equivalence. Supplementary File S1: Completed PRISMA 2020 checklist mapped to the final JCM submission. Supplementary File S2: Search strategy and study-selection documentation, including verified source accounting, retained database-specific search strategies, execution-history limitations, update/completeness provenance, and final screening-closure documentation. Supplementary File S3: Reviewer agreement audit for the final *N* = 270 review, including the paired eligibility-decision audit, data-extraction discrepancy audit, critical-appraisal agreement contingency table, and disagreement-resolution records.

## Abbreviations

AI	artificial intelligence
AMD	age-related macular degeneration
AUC	area under the curve
AUROC	area under the receiver operating characteristic curve
AUPRC	area under the precision–recall curve
CAM	class activation mapping
CNN	convolutional neural network
DL	deep learning
DME	diabetic macular oedema
DR	diabetic retinopathy
GRADE	Grading of Recommendations Assessment, Development and Evaluation
IRD	inherited retinal disease
JBI	Joanna Briggs Institute
LLM	large language model
MCC	Matthews correlation coefficient
ML	machine learning
nAMD	neovascular age-related macular degeneration
OCT	optical coherence tomography
OCTA	optical coherence tomography angiography
PRISMA	Preferred Reporting Items for Systematic Reviews and Meta-Analyses
QUADAS-2	Quality Assessment of Diagnostic Accuracy Studies 2
RNFL	retinal nerve fiber layer
RoB 2	Risk of Bias 2
ROBINS-I	Risk Of Bias in Non-randomized Studies of Interventions
ROP	retinopathy of prematurity
SHAP	SHapley Additive exPlanations
vCDR	vertical cup-to-disc ratio
VEGF	vascular endothelial growth factor
VF	visual field
VLM	vision-language model
XAI	explainable artificial intelligence
XRAI	eXtended Region-based Attribution Integration
