# Supplementary material for: Artificial Intelligence in Eye Disease: A Systematic Review of Screening, Diagnosis, Prognosis, Treatment-Response Prediction, and Clinical Decision Support
Source: J Clin Med. 2026 Oct 14;15(20):7957. doi: 10.3390/jcm15207957 (PMC13706016; doi:10.3390/jcm15207957)
Supplement: Supplementary file 1 [file jcm-15-07957-s001.zip › Supplementary File S2.pdf]

Supplementary File S2. Search Strategy and Study Selection

PROSPERO CRD420251241901 | Final included evidence base: N = 270

S4.1. Search Window and Information Sources

Eligible English-language literature first published from 1 January 2015 through 10 August 2026 was eligible. The same clinical-function eligibility framework was used across the completed evidence base. The lower boundary was an operational post-registration criterion documented in review materials before manuscript submission. The final publication-eligibility cutoff was 10 August 2026; this was a cutoff for first-publication eligibility and was not a synchronized execution date for every database. The historical identification denominator reconciles exactly source by source. Six database sources contributed 13,789 records: PubMed/MEDLINE 3455; Embase 2290; Scopus 1671; Web of Science 1000; Cochrane Library/CENTRAL 800; and IEEE Xplore 4573. Other-method identification contributed 17 records: Google Scholar 10 and citation/reference-list/manual searching 7. These components sum exactly to 13,806. The database-specific search strings themselves are retained in the review documentation and reproduced below. A native database-generated Search History/export, exact platform identifier, controlled-vocabulary execution log, or exact execution date is not retained for every source; where such execution-history metadata are unavailable, that limitation is stated explicitly without implying that the search strategy itself is unavailable.

PROSPERO CRD420251241901 Version 1.0 was published on 29 November 2025 and did not specify a publication-date restriction. During final protocol documentation, the public record was aligned with the operational eligibility window already used by the review. Version 2.0 was published on 25 August 2026 and records the 1 January 2015–10 August 2026 window. This registry alignment occurred after the completed search/screening work and did not change the core clinical question or introduce a result-dependent eligibility rule.

S4.2. Database-Specific Search Strategies

Search management and screening were conducted using Covidence (Veritas Health Innovation, Melbourne, Australia; https://www.covidence.org/). Table S4.1 reproduces the retained database-specific search strategies and reports the verified historical source counts, field/interface syntax where documented, eligibility limits, and retained execution/import timing. The retained search strings are distinguished from native database-generated Search History/export metadata: where a native export, exact platform identifier, or exact execution timestamp is not preserved, this is stated explicitly. The table reports the locked publication-eligibility limit separately from retained execution/import timing and does not imply that every source was synchronously rerun on 10 August 2026.

Historical supplementary identification comprised Google Scholar (n=10) and citation/reference-list/manual searching (n=7). Search-sensitivity/completeness checks for less explicitly represented conditions used the same clinical-function eligibility framework and included targeted reference/citation review for inherited retinal diseases, pediatric eye disease, and optic-nerve/glaucoma-related terminology. Publisher-direct/current-awareness routes were also used during later completeness work where retained; AMEDEO/British Journal of Ophthalmology is treated as such a supplementary current-awareness route, not as a seventh database. After the historical identification ledger was established, iterative update/completeness work continued toward the locked publication cutoff. The formal post-November update log recorded 20 candidate reports and 11 eligible original-study additions and preserves a study-level source URL for each included report. Later overlapping

retrieval/deduplication did not preserve a unique originating database for every candidate, so database-specific update hit counts are not reconstructed and current indexing is not used to infer historical origin.

Source / platform	Documented strategy / approach	Eligibility limits / documented run timing	Archive status / source count
PubMed/MEDLINE	((artificial intelligence OR machine learning OR deep learning OR neural network* OR algorithm* OR large language model* OR AI) AND (ophthalmolog* OR optometr* OR ocular OR eye OR retina* OR glaucoma OR diabetic retinopathy OR macular degeneration OR optical coherence tomography OR OCT OR fundus OR strabismus OR amblyopia OR retinopathy of prematurity) AND (diagnos* OR screen* OR classific* OR segment* OR prognos* OR predict* OR treatment response OR decision support OR triage OR referral OR management))	Eligibility: English-language first publication 1 Jan 2015–10 Aug 2026. Retained primary import date: 30 Nov 2025; later update/completeness work documented separately.	PubMed/MEDLINE. Verified historical primary import n = 3455 (retained import date 30 Nov 2025). Boolean strategy is retained in the review documentation. Later additions are not retrospectively assigned to PubMed unless explicit source provenance is preserved.
Embase	(artificial intelligence OR machine learning OR deep learning OR neural network* OR algorithm* OR large language model* OR AI) AND (ophthalmolog* OR optometr* OR ocular OR eye OR retina* OR glaucoma OR diabetic retinopathy OR macular degeneration OR optical coherence tomography OR OCT OR fundus OR strabismus OR amblyopia OR retinopathy of prematurity) AND (diagnos* OR screen* OR classific* OR segment* OR prognos* OR predict* OR treatment response OR decision support OR triage OR referral OR management); title/abstract/keyword fields and Emtree-equivalent subject headings were used where available.	Eligibility: English-language first publication 1 Jan 2015–10 Aug 2026. Retained primary import date: 27 Nov 2025; no authenticated synchronized final rerun export retained.	Embase. Historical source count n = 2290 (retained import date 27 Nov 2025). The database-specific Embase Boolean/Emtree search strategy is retained and reproduced in this table. The exact platform identifier and native database-generated Search History/export are not retained; this execution-history limitation is distinct from availability of the search string.
Scopus	TITLE-ABS-KEY(("artificial intelligence" OR "machine learning" OR "deep learning" OR "neural network*" OR algorithm* OR "large language model*" OR AI) AND (ophthalmolog* OR optometr* OR ocular OR eye OR retina* OR glaucoma OR "diabetic retinopathy" OR "macular degeneration" OR "optical coherence tomography" OR OCT OR fundus OR strabismus OR amblyopia OR "retinopathy of prematurity") AND (diagnos* OR screen* OR classific* OR segment* OR prognos* OR predict* OR "treatment response" OR "decision support" OR triage OR referral OR management))	Eligibility: English-language first publication 1 Jan 2015–10 Aug 2026. Retained primary import date: 30 Nov 2025; later source-specific update attribution not safely recoverable after overlap/deduplication.	Scopus. Verified historical primary import n = 1671 (retained import date 30 Nov 2025). TITLE-ABS-KEY syntax is documented. Later unique source attribution was not preserved after overlapping retrieval/deduplication.
Web of Science	TS=("artificial intelligence" OR "machine learning" OR "deep learning" OR "neural network*" OR algorithm* OR "large	Eligibility: English-language first publication 1 Jan 2015–	Web of Science. Historical source count n = 1000. The documented Topic-field

	language model*" OR AI) AND TS=(ophthalmolog* OR optometr* OR ocular OR eye OR retina* OR glaucoma OR "diabetic retinopathy" OR "macular degeneration" OR "optical coherence tomography" OR OCT OR fundus OR strabismus OR amblyopia OR "retinopathy of prematurity") AND TS=(diagnos* OR screen* OR classific* OR segment* OR prognos* OR predict* OR "treatment response" OR "decision support" OR triage OR referral OR management)	10 Aug 2026. Search executed as part of the original workflow. Historical source count n = 1000 is retained in the source-accounting documentation; the native historical search export and exact final execution date are not retained. 10 Aug is the publication cutoff, not a claimed synchronized run date.	(TS=) search strategy is retained and reproduced in this table. The native database-generated search-history export and exact execution date are not retained and are not reconstructed from current indexing.
Cochrane Library	("artificial intelligence" OR "machine learning" OR "deep learning" OR "neural network*" OR algorithm* OR "large language model*" OR AI) AND (ophthalmology OR optometry OR ocular OR eye OR retina OR glaucoma OR "diabetic retinopathy" OR "macular degeneration" OR "optical coherence tomography" OR OCT OR fundus OR strabismus OR amblyopia OR "retinopathy of prematurity") AND (diagnosis OR screening OR classification OR segmentation OR prognosis OR prediction OR "treatment response" OR "decision support" OR triage OR referral OR management)	Eligibility: English-language first publication 1 Jan 2015–10 Aug 2026. Cochrane Library/CENTRAL search executed as part of the original workflow. Historical source count n = 800 is retained in the source-accounting documentation; the native historical Search Manager export and exact final execution date are not retained.	Cochrane Library/CENTRAL. Historical source count n = 800. The documented Boolean concept-block search strategy is retained and reproduced in this table. The native Search Manager/export and exact execution date are not retained and are not reconstructed from current indexing.
IEEE Xplore	(("All Metadata":"artificial intelligence" OR "All Metadata":"machine learning" OR "All Metadata":"deep learning" OR "All Metadata":"neural network" OR "All Metadata":algorithm OR "All Metadata":"large language model") AND ("All Metadata":ophthalmology OR "All Metadata":optometry OR "All Metadata":ocular OR "All Metadata":retina OR "All Metadata":glaucoma OR "All Metadata":"diabetic retinopathy" OR "All Metadata":"optical coherence tomography" OR "All Metadata":fundus) AND ("All Metadata":diagnosis OR "All Metadata":screening OR "All Metadata":classification OR "All Metadata":segmentation OR "All Metadata":prognosis OR "All Metadata":"decision support"))	Eligibility: English-language first publication 2015–10 Aug 2026. Retained primary import date: 30 Nov 2025; later unique source attribution not preserved after overlapping retrieval/deduplication.	IEEE Xplore. Verified historical primary import n = 4573 (retained import date 30 Nov 2025). All-Metadata syntax is documented. Later unique source attribution was not preserved after overlapping retrieval/deduplication.
Supplementary sources	Google Scholar supplementary searching and citation/reference-list/manual searching were used in	Historical supplementary identification n = 17:	The historical supplementary subtotal of 17 is included within the

	the historical identification phase. Publisher-direct retrieval/current-awareness routes and other iterative completeness methods were used later where retained; AMEDEO/British Journal of Ophthalmology is treated as a supplementary current-awareness route, not as a database.	Google Scholar n = 10; citation/reference-list/manual searching n = 7. Later publisher-direct/current-awareness and completeness routes were applied toward the fixed 10 Aug 2026 publication cutoff and are documented at report/study level where provenance is retained.	13,806-record identification denominator. Formal 11-study update source provenance is reproduced in Supplementary Data S1. Later record-level routes are assigned only when explicitly retained; current indexing does not establish historical origin.

S4.3. Consolidated Study Selection

Final consolidated PRISMA accounting. The verified identification denominator contains 13,806 records, comprising 13,789 database records and 17 supplementary records, followed by 1044 duplicate removals and 12,762 records screened. After final screening closure under the locked 10 August 2026 publication window, 12,273 records were excluded at title/abstract screening and 489 reports were sought for retrieval. Three reports were not retrieved, leaving 486 reports assessed for full-text eligibility. At full text, 216 reports were excluded—104 not ophthalmology/optometry, 20 ineligible clinical AI function, 30 ineligible outcome, 56 duplicate/overlapping reports, and 6 with insufficient clinical applicability—yielding 270 included studies. These counts are reported in Figure 1.

Consolidated selection item	n	Interpretation
Records identified	13,806	Exact source reconciliation: database subtotal 13,789 = PubMed/MEDLINE 3455 + Embase 2290 + Scopus 1671 + Web of Science 1000 + Cochrane Library/CENTRAL 800 + IEEE Xplore 4573; supplementary subtotal 17 = Google Scholar 10 + citation/reference-list/manual searching 7; overall total = 13,806.
Duplicates removed	1044	Removed before title/abstract screening.
Records screened	12,762	Title/abstract screening.
Records excluded	12,273	Title/abstract exclusions after final screening closure.
Reports sought for retrieval	489	Potentially eligible full-text reports sought in the finalized screening flow.
Reports not retrieved	3	F001, F004 and F014 remained not retrieved.
Reports assessed for eligibility	486	Full-text eligibility assessment in the finalized screening flow.
Full-text reports excluded	216	Not ophthalmology/optometry (n=104); ineligible clinical AI function (n=20); ineligible outcome (n=30); duplicate/overlapping report (n=56); insufficient clinical applicability (n=6).
Final included evidence base	270	Unique original studies included in the qualitative synthesis; 486 - 216 = 270.
Final screening-closure audit	18	Fourteen records previously excluded under the superseded date-only criterion plus four unresolved/not-retrieved reports were rechecked to finalize the counts.
Eligible records incorporated during final closure	15	All 14 date-only records were eligible; F008 became retrievable and eligible.
Still not retrieved after final closure	3	F001, F004 and F014 remained without retrievable full reports and were not extracted/appraised.

Final evidence base	270	Unified final PRISMA endpoint. Verified raw identification denominator remains 13,806.

Audit trail for final screening closure. Eighteen previously identified records in the review dataset required final disposition: 14 reports previously excluded under the superseded 31 May 2026 date-only criterion plus four previously unresolved/not-retrieved reports. The 14 date-only records were re-adjudicated under the unchanged clinical-function criteria and the locked 10 August 2026 publication window; all 14 were eligible. Of the four unresolved reports, F008 became fully retrievable and eligible, while F001, F004, and F014 remained not retrieved and therefore remained outside extraction and appraisal. These records were part of the existing screening universe rather than new raw database hits. Accordingly, the final PRISMA counts were consolidated to 12,273 title/abstract exclusions, 489 reports sought, 3 not retrieved, 486 assessed, 216 full-text exclusions, and N=270 included, while the verified 13,806 identification denominator remained unchanged. This was a record-level screening/cutoff closure, not a synchronized native rerun of the six databases. Study-level routes are reported only where retained; F014 remains documented as an AMEDEO/British Journal of Ophthalmology supplementary current-awareness route.

S4.4. Clinical-Function Eligibility Rule

Eligible original studies evaluated an AI system in eye care and addressed at least one clinically relevant function: screening/detection/triage, diagnosis/classification/grading, prognosis/risk prediction, treatment planning or treatment-response prediction, monitoring/follow-up, workflow/implementation, or clinician-facing decision support/management. Each included study was assigned one primary clinical-area category based on its dominant disease target/clinical question; multifaceted clinical functions were coded non-mutually exclusively. Glaucoma-targeted optic-nerve studies were classified as Glaucoma, whereas non-glaucomatous optic-disc/neuro-ophthalmic studies remained in the neuro-ophthalmology/optic-nerve category.

S4.5. Protocol Updates and Methodological Clarifications

Protocol item	Initial registered plan	Completed review / amendment	Timing and rationale
Publication-date restriction	No date restriction stated in Version 1.0 (29 Nov 2025).	Operational eligibility window 1 Jan 2015–10 Aug 2026; Version 2.0 published 25 Aug 2026.	The 2015 boundary was documented in review materials before manuscript submission; the public registry was aligned during final protocol-to-manuscript screening closure after search/screening completion. It was not selected in response to study results.
Critical appraisal	QUADAS-2, RoB 2 and ROBINS-I listed in the original record.	Design-mapped JBI/adapted-JBI item-level critical appraisal for the heterogeneous final corpus.	The eligible design mix included diagnostic/model-validation, prognostic/cohort, cross-sectional/content-evaluation, implementation and randomized studies. The framework was finalized after the design mix was known and before full-corpus appraisal reporting; appraisal did not determine eligibility. Parallel use of several non-interchangeable instruments was not selected because it would fragment the corpus into subset-specific outputs and would not solve the poor fit of classic QUADAS-2 assumptions for many AI model-development/validation studies.

Quantitative synthesis	Meta-analysis conditional on sufficient homogeneity.	Narrative synthesis; no statistical pooling.	The protocol condition for pooling was not met because of marked heterogeneity in tasks, populations, modalities, thresholds, comparators and outcomes.

Search-history limitation: the retained archive and source-accounting documentation support exact historical source counts that reconcile to 13,806 records: 13,789 from six database sources and 17 from other-method identification. The database-specific search strings are retained/documented and reproduced in Table S4.1. What is not retained for every source is the native database-generated Search History/export and, for some sources, the exact platform identifier or exact execution timestamp. Those execution-history limitations are not reconstructed from current indexing and should not be interpreted as absence of the underlying search strategy. For later update/completeness work, overlapping retrieval and deduplication did not preserve a unique originating database for every candidate, so database-specific update hit counts are not retrospectively assigned. The 10 August 2026 date is therefore reported as the locked publication-eligibility cutoff, not as a synchronized execution date for every source. The historical other-method count of 17 refers specifically to Google Scholar (10) and citation/reference-list/manual searching (7); later publisher-direct/current-awareness provenance, including F014 through AMEDEO/British Journal of Ophthalmology, is reported at report/study level and is not added again to the 13,806-record denominator.

These methodological updates did not change the core clinical review question. The design-specific appraisal framework was selected after the eligible design mix was known and before the finalized full-corpus appraisal was reported; appraisal judgments were not used to determine study eligibility. The manuscript reports item-level JBI/adapted-JBI judgments rather than an unvalidated global lower/some/high-concern score. The design-mapped layer was retained across the heterogeneous corpus because parallel application of several instrument-specific tools would generate subset-specific outputs that are not directly interchangeable and would still not fit many AI model-development/validation reports. Quantitative pooling was not performed because the protocol-specified condition of sufficient clinical and methodological homogeneity was not met.
