# Supplementary material for: Artificial Intelligence in Eye Disease: A Systematic Review of Screening, Diagnosis, Prognosis, Treatment-Response Prediction, and Clinical Decision Support
Source: J Clin Med. 2026 Oct 14;15(20):7957. doi: 10.3390/jcm15207957 (PMC13706016; doi:10.3390/jcm15207957)
Supplement: Supplementary file 1 [file jcm-15-07957-s001.zip › Supplementary File S1.pdf]

Supplementary File S1. PRISMA 2020 Checklist

Manuscript title: Artificial Intelligence in Eye Disease: A Systematic Review of Screening, Diagnosis, Prognosis, Treatment-Response Prediction, and Clinical Decision Support

PROSPERO registration: CRD420251241901

This checklist is mapped to the final manuscript and documents PRISMA 2020 reporting across the final evidence base of 270 included studies, including performance ranges, clinical-function/year analyses, final disease-category and design classifications, stage-specific reviewer-agreement audits, item-level appraisal reporting, validation/XAI definitions, scope coding, retained historical search information, update/completeness routes, and the final PRISMA screening counts with separate database and other-method identification streams.

PRISMA item	Topic	Checklist detail / how addressed	Location in manuscript or supplement
1	Title	Identify the report as a systematic review.	Title page, p. 1
2	Abstract	Provide a structured summary with Background/Objectives, Methods, Results, and Conclusions.	Structured Abstract, p. 1
3	Rationale	Explain the rationale and novelty of the broad clinical-pathway synthesis relative to prior ophthalmic-AI reviews.	Introduction, p. 2; Table 5, pp. 18–19
4	Objectives	State objectives across screening, diagnosis/classification, prognosis, treatment response, monitoring, triage/referral, decision support, validation, and explainability.	End of Introduction, pp. 2–3
5	Eligibility criteria	Specify original-research eligibility, one-primary-clinical-area rule, non-mutually-exclusive clinical-function coding, pediatric definition, optometry handling, date/language limits, comparator definition, and IRD eligibility.	Methods 2.3–2.4, pp. 4–6; Supplementary Data S1, sheet “Coding_Codebook”
6	Information sources	List information sources, eligibility/search cutoff, source-specific historical counts, retained search documentation, and any execution-history limitations.	Methods 2.2, pp. 3–4; Supplementary File S2
7	Search strategy	Provide the retained database-specific search strategies/field syntax, controlled/free-text terms, limits, exact historical source counts, retained execution/import timing, and clearly distinguish the documented search strings from any native database-generated Search History/export metadata that are not retained.	Supplementary File S2, Table S4.1
8	Selection process	Describe independent two-reviewer screening and discrepancy resolution; report the retained final-study eligibility audit (260/270 concordant decisions; 96.3% observed agreement) with the explicit limitation that this audit does not represent all 12,762 title/abstract records.	Methods 2.4, pp. 4–6; Supplementary Data S1; Supplementary File S3; Figure 1 and caption
9	Data collection process	Describe independent duplicate data extraction by two reviewers, structured fields, source verification and discrepancy resolution; report complete study-level concordance for 265/270 studies (98.1%) and eight field-level discrepancies across five studies, all resolved.	Methods 2.4, pp. 5–6; Supplementary Data S1; Supplementary File S3

PRISMA item	Topic	Checklist detail / how addressed	Location in manuscript or supplement
10a	Data items - outcomes	Define all outcomes/domains sought, including performance, workflow/efficiency, reliability, usability, trust, safety/harm, validation, comparators, and XAI.	Methods 2.4–2.6, pp. 5–7; Supplementary Data S1, sheet “Coding_Codebook”
10b	Data items - other variables	Record disease/clinical area, AI method/architecture, modality, dataset/sample, source type, center structure, validation, comparator/reference standard, year, identifiers, and assumptions; unsupported fields were not imputed.	Methods 2.4, pp. 5–6; Supplementary Data S1
11	Study risk of bias / quality assessment	Describe independent two-reviewer design-mapped JBI/adapted-JBI item-level critical appraisal, 55-study calibration, disagreement resolution, N/A as design-inapplicable, adaptation limitations, and the retained agreement audit (2711/2717 applicable judgments; 99.8%; Cohen's kappa = 0.995).	Methods 2.5, p. 6; Results 3.5; Supplementary Table S1; Supplementary File S3
12	Effect measures	Extract study-reported metrics (e.g., sensitivity, specificity, AUC, accuracy, F1, kappa/ICC, MAE, calibration, overlap metrics) without inappropriate conversion or pooling.	Methods 2.4–2.6, pp. 5–7; Results 3.4, pp. 13–14
13a	Synthesis methods - study eligibility	Describe narrative synthesis, unpooled descriptive performance ranges covering all 13 primary clinical areas, function-by-year counts, disease/category audits, validation/XAI strata, focused general-purpose LLM/VLM audit, and the decision not to pool heterogeneous data.	Methods 2.6, pp. 6–7; Supplementary Data S1 analysis sheets
13b	Synthesis methods - data preparation	Describe methods used to prepare data for presentation, including one-primary-disease assignment, non-mutually-exclusive function coding, and conservative extraction of explicit performance values.	Methods 2.4–2.6, pp. 5–7; Supplementary Data S1, sheet “Coding_Codebook”
13c	Synthesis methods - tabulation/visual display	Present PRISMA flow, publication-year trend, primary AI-method distribution, validation-design categories, compact evidence-base summary, disease × method × modality × outcome synthesis, performance ranges, function-by-year matrix, and search/provenance documentation.	Figures 1–4, pp. 9–14; Tables 1–5, pp. 12–19; Supplementary Data S1
13d	Synthesis methods - statistical/synthesis methods	Use descriptive counts and narrative synthesis; no meta-analysis because clinical and methodological homogeneity was insufficient.	Methods 2.6, pp. 6–7; Results 3.2–3.6, pp. 10–17
13e	Synthesis methods - heterogeneity exploration	Describe methods used to explore heterogeneity and clinical translation: disease, function, modality, validation type, comparator status, XAI, pediatric/optometry scope, and dataset reuse.	Methods 2.6, pp. 6–7; Results 3.2–3.6, pp. 10–17
13f	Synthesis methods - sensitivity analyses	Not applicable: no pooled effect estimate or meta-analysis was conducted; therefore, formal meta-analytic sensitivity analyses were not performed.	Methods 2.6, pp. 6–7

PRISMA item	Topic	Checklist detail / how addressed	Location in manuscript or supplement
14	Reporting bias assessment	No formal statistical reporting-bias assessment was undertaken because of heterogeneous narrative evidence; this is stated explicitly and interpreted as a review limitation.	Methods 2.6, p. 7; Discussion 4.5, p. 21
15	Certainty assessment	No formal GRADE/certainty rating was applied across heterogeneous designs; certainty/transferability is discussed using item-level appraisal, validation type, comparator status, and reporting limitations.	Methods 2.6, p. 7; Discussion 4.5, p. 21
16a	Study selection results	Describe one final PRISMA flow with separate identification streams: databases n=13,789 and other methods n=17 (total n=13,806), followed by 1044 duplicates removed, 12,762 screened, 12,273 title/abstract exclusions, 489 reports sought, 3 not retrieved, 486 assessed, 216 full-text exclusions, and N=270 included. The audit trail used to finalize these counts is retained in S1/S4; the 15 eligible records finalized during screening closure are not treated as new raw database hits.	Results 3.1, pp. 7–8; Figure 1 and caption, p. 9; Supplementary Data S1 sheets “Search_Chronology” and “Final_Screening_Closure_18”; Supplementary File S2
16b	Excluded studies	Report all 216 full-text exclusion categories and identify the three potentially eligible reports that remained not retrieved (F001, F004, F014).	Results 3.1, pp. 7–8; Supplementary File S2
17	Study characteristics	Cite study-level characteristics and provide the complete N=270 structured evidence table, including final primary clinical-area assignments, references 269–283 for the 15 records incorporated during final screening closure, and coding/audit sheets.	Results 3.2–3.4, pp. 10–14; Supplementary Data S1
18	Risk-of-bias / appraisal results	Report item-level critical appraisal, final design-family distribution (200 diagnostic/model-validation, 48 cohort/prognostic, 13 cross-sectional/content evaluation, 5 quasi-experimental/implementation, 4 randomized/interventional), and appraisal inter-reviewer agreement (2711/2717 applicable judgments; 99.8%; Cohen's kappa = 0.995).	Results 3.5, p. 16; Supplementary Table S1; Supplementary File S3
19	Results of individual studies	Report descriptive results including disease-specific unpooled AUC/sensitivity/specificity ranges for all primary areas, clinical-function counts/year trends, focused general-purpose LLM/VLM comparisons, targeted outcome-domain counts, validation type, comparator status and XAI categories.	Results 3.2–3.6, pp. 10–17; Supplementary Data S1 analysis sheets
20a	Results of syntheses - contributing studies	Present results of each synthesis and explicitly distinguish evidence amount from evidence strength and technical from clinically independent validation.	Results 3.2–3.6, pp. 10–17; Discussion 4.1–4.3, pp. 17–21
20b	Results of syntheses - statistical results	Report descriptive counts/percentages and patterns; pooled effect estimates and statistical heterogeneity are not applicable because meta-analysis was not performed.	Results 3.2–3.6, pp. 10–17
20c	Results of syntheses - heterogeneity	Report disease-, model-, modality-, validation-, comparator-, advanced-metric-, and XAI-specific evidence-density patterns and discuss their clinical interpretation.	Results 3.3–3.6, pp. 12–17; Discussion 4.3, pp. 19–21

PRISMA item	Topic	Checklist detail / how addressed	Location in manuscript or supplement
20d	Results of syntheses - sensitivity analyses	Not applicable because no pooled quantitative synthesis was performed.	Methods 2.6, pp. 6–7
21	Reporting biases	Discuss incomplete reporting of calibration, subgroup/equity analyses, implementation outcomes, external validation, and source-depth variation.	Discussion 4.3–4.5, pp. 19–21; Supplementary Table S1
22	Certainty of evidence	Discuss limitations to confidence/generalizability from heterogeneity, incomplete independent validation, few direct clinical comparators, source-depth variation, and methodological concerns.	Discussion 4.3–4.5, pp. 19–21
23a	Discussion - interpretation	Interpret findings in relation to clinical translation from diagnosis to management and recent ophthalmic-AI literature.	Discussion 4.1–4.4, pp. 17–21
23b	Discussion - limitations of evidence	Discuss limitations of the evidence, including heterogeneity, sparse safety/usability/trust outcomes, dataset reuse, validation quality and generalizability.	Discussion 4.3–4.5, pp. 19–21
23c	Discussion - limitations of review processes	Discuss review-process limitations: post-registration amendments, pragmatic adapted appraisal, incomplete retention of some native search-execution/export metadata, final screening closure under the locked cutoff, three still-not-retrieved reports, the limited scope of the retained final-study eligibility agreement audit (not all 12,762 title/abstract records), no meta-analysis, no formal reporting-bias test/GRADE rating, and English-language restriction.	Discussion 4.5, p. 21; Supplementary File S2; Supplementary File S3
23d	Discussion - implications	Describe research and clinical implications, including prospective external validation, clinically meaningful treatment/management endpoints, XAI evaluation, and implementation.	Discussion 4.6, pp. 21–22; Conclusions, p. 22
24a	Registration	Report PROSPERO registration CRD420251241901.	Title page, p. 1; Methods 2.1, p. 3
24b	Protocol availability	Identify the public PROSPERO registration record.	Methods 2.1, p. 3; PROSPERO CRD420251241901
24c	Protocol amendments	Report PROSPERO Version 1.0 and Version 2.0 timing, the publication-window/appraisal amendments, and the final screening closure needed to apply the locked 10 August 2026 publication cutoff consistently across retained records; explain that the clinical eligibility criteria did not change.	Methods 2.2, pp. 3–4; Methods 2.5–2.6, pp. 6–7; Supplementary File S2
25	Support	Report funding source and role of funder.	Funding, p. 23
26	Competing interests	Report conflicts of interest.	Conflicts of Interest, p. 23

PRISMA item	Topic	Checklist detail / how addressed	Location in manuscript or supplement
27	Availability of data, code, and materials	Describe availability of the complete N=270 evidence workbook, N=270 item-level JBI matrix, PRISMA checklist, and search/provenance/final-screening documentation.	Supplementary Materials, pp. 22–23; Data Availability Statement, p. 23; Supplementary Data S1; Supplementary Table S1; Supplementary Files S1–S3
